# Factors affecting implementation of patient-reported outcome and experience measures in a pediatric health system

**DOI:** 10.1186/s41687-023-00563-1

**Published:** 2023-03-09

**Authors:** Erin McCabe, Sarah Rabi, Sumedh Bele, Jennifer D. Zwicker, Maria J. Santana

**Affiliations:** 1 School of Public Policy, University of Calgary, Calgary, Canada; 2 Department of Pediatrics, University of Calgary, Calgary, Canada; 3 Department of Community Health Sciences, University of Calgary, Calgary, Canada; 4 Patient Engagement Team, Alberta Strategy for Patient-Oriented Research Support Unit, Calgary, Canada; 5 Faculty of Kinesiology, University of Calgary, Calgary, Canada

**Keywords:** Patient-reported outcome measures, Patient-reported experience measures, Pediatric, Implementation, Health measurement, Value-based health care

## Abstract

**Background:**

The use of patient-reported outcome measures (PROMs) and patient-reported experience measures (PREMs) in pediatric clinical practice can enhance clinical care and bring children and families’ perspectives into evaluations of healthcare services. Implementing these measures is complex and requires a thorough assessment of the context of implementation The purpose of this study is to describe the barriers and facilitators to PROMs and PREMs implementation and to recommend strategies for implementing these measures in a pediatric health system.

**Methods:**

We used a qualitative descriptive approach to analyse data from interviews to understand the experiences of PROMs and PREMs users across different pediatric settings in a single Canadian healthcare system.

**Results:**

There were 23 participants representing a variety of roles within the healthcare system and pediatric populations. We found five main factors that affected implementation of PROMs and PREMs in pediatric settings: 1) Characteristics of PROMs and PREMs; 2) Individual’s beliefs; 3) Administering PROMs and PREMs; 4) Designing clinical workflows; and 5) Incentives for using PROMs and PREMs. Thirteen recommendations for integrating PROMs and PREMs in pediatric health settings are provided.

**Conclusions:**

Implementing and sustaining the use of PROMs and PREMs in pediatric health settings presents several challenges. The information presented will be useful for individuals who are planning or evaluating the implementation of PROMs and PREMs in pediatric settings.

**Supplementary Information:**

The online version contains supplementary material available at 10.1186/s41687-023-00563-1.

## Abstract (highlights)

Patient-reported outcome measures are standardized questionnaires that ask patients about their health and well-being and are useful for tracking patient progress and outcomes of care. Patient-reported experience measures ask patients about their experiences while receiving care and are useful for quality improvement and experience research. Clinicians can use them to help identify patients’ needs, monitor a person’s health status, and to give extra information that helps with planning treatment. These measures also provide data that health services can use to understand whether their programs are helpful from the patients’ perspective. We know that using these measures is beneficial, but there are many challenges to overcome before they are used consistently in a health system. In pediatrics there are special considerations, like whether a caregiver or child should be answering the questions, or whether a parent should be able to see their child’s answers. In this study, we interviewed people in the pediatric health system who are successfully using patient-reported outcome and experience measures about the challenges they face and the strategies they find helpful for using these measures. This information will be helpful for people who are planning to start using these measures in pediatric health care.

**Supplementary Information:**

The online version contains supplementary material available at 10.1186/s41687-023-00563-1.

## Background

Providing patient- and family-centred care (PFCC) is a priority in health systems internationally [1, 2]. This aligns closely with recent advocacy for the Quadruple Aim Framework, which simultaneously pursues improvement in patient experience, health care provider experience, population health, and reduced costs [3]. Patient-reported outcome measures (PROMs) and patient-reported experience measures (PREMs) are an approach to enhancing PFCC, extending the boundaries of patient care beyond the confines of the traditional healthcare system [4]. PROMs are standardized and validated questionnaires filled out by the patient about their health status or well-being [5]. Using PROMs in clinical practice may enhance health outcomes, communication, and helps clinicians tailor treatment plans to a patient’s self-identified needs and goals [5–7]. Data generated by PROMs is also useful for research aimed at understanding disease trajectories and treatment effectiveness, and for evaluating efficiency of healthcare services and quality improvement [5]. Alternatively, PREMs ask the patient about their experiences of care and are most commonly used to inform program evaluation and quality improvement efforts, but may also be used in clinical practice [8, 9]. The concurrent use of PROMs and PREMs in patient care therefore allows professionals to more comprehensively capture the patient perspective [10].

In pediatrics, PROMs and PREMs have proven beneficial for deepening patient-clinician relations, improving patient health outcomes, and driving patient care innovation [11–13]. While they are often used in pediatrics, incorportating PROMs and PREMs systematically into pediatric health systems is rare [11–13]. This may be due in part to concerns of reduced patient capacity to meaningfully contribute to their care, as well as the use of PROMs and PREMs in pediatric care has been traditionally limited [2]. However, lack of consistent use of these patient-centered measures in pediatric settings is restricting the ability of health teams to gauge the care priorities of children and adolescents, and incorporate the patient perspective into evaluations of health services [2, 14]. Therefore, there is a need to further explore and understand approaches for implementing PROMs and PREMs into pediatric healthcare services. These measures are key to infom the Quadruple Aim Framework and promote the practice of PFCC.

Implementation research is the study of methods to promote the systematic uptake of evidence-based practices into routine clinical care [15]. Implementation science is important for successful changes in healthcare systems, providing a mechanism to operationalize health innovation efforts [16]. Using implementation science will ease the integration of PROMs and PREMs into complex health systems and to address foreseeable barriers prior to implementation [17]. Guidelines exist to guide PROM and PREM implementation into clinical practice [18, 19]. There is also guidance, mainly from adult settings, on how to apply implementation science frameworks to PROMs and PREMs integration [17, 20–22]. However, this research cannot be directly applied to pediatric populations as unique considerations exist, from the design of the measures through to their application in clinic. There is limited research to guide pediatric PROM and PREM implementation in pediatric settings.

An important step in designing for implementation is a thorough assessment of barriers and facilitators [23]. Therefore, the purpose of this study was to describe the barriers and facilitators to implementing PROMs and PREMs across pediatric settings in a large health system. This study will help administrators when constructing a system-level implementation strategy for PROMs and PREMs in pediatric health settings. Our specific objectives were to: (1) identify the factors that affect the successful integration of PROMs and PREMs in a pediatric health system, and (2) recommend strategies for implementation.

## Methods

### Design

In this study we employed a qualitative descriptive approach to understand the experiences of PROMs and PREMs users across different pediatric settings in a single Canadian province. Qualitative description studies offer a comprehensive summary of an event or phenomenon, which has a low degree of interpretation of the data and is good for applied research meant for practitioners and policy-makers [24].

### Setting and participants

This study was conducted in Alberta, Canada. Alberta is Canada’s fourth largest province and has the country’s first province-wide healthcare system called Alberta Health Services (AHS). The pediatric health system includes two large tertiary care hospitals and one pediatric tertiary rehabilitation hospital. In this study, we wanted to capture a broad overview of factors affecting the use of PROMs and PREMs in pediatrics in Alberta. We therefore targeted any individual familiar with the use of PROMs and PREMs in any pediatric health setting (i.e., inpatient, outpatient, and community), working in the health system at the micro (clinical), meso (clinical or program) and macro (health system) level. This included health care providers, academic clinical and health systems researchers, non-academic evaluation and research specialists, and administrators.

### Data collection

This study was based on combined semi-structured interview data from two sources: (1) secondary analysis of semi-structured interview data obtained in a previous mixed methods study [13]; and 2) semi-structured interview data collected as part of an environmental scan with the aim of preparing a strategy to integrate PROMs and PREMs into pediatric care across Alberta. Data from Study 1 contained more perspectives from academic clinical researchers while Study 2 included a greater proportion of system-level perspectives (i.e., evaluation specialists and administrators). Combining these two datasets allowed us to gain a more comprehensive overview of the factors that could affect successful implementation of PROMs and PREMs users in Alberta. A similar semi-structured interview guide was used for Study 1 and 2, with some modifications in Study 2 to target participant perspectives on facilitators and barriers to PROM and PREM use (see Additional file 1 and Additional file 2).

*Study 1*. See [blinded for peer review] for a detailed account of the study, however, briefly, data were collected between May 2021 and April 2022 from 14 individuals who were PROMs and PREMs users in Alberta (7 physicians, 1 psychologist, 6 academic researchers). The focus of the qualitative arm was to understand the uses, benefits, and challenges associated with PROMs and PREMs in pediatric settings.

*Study 2*. Participants were recruited through newsletters and emails of professional groups (e.g., health professional associations, primary care networks, pediatric research institutions). Potential participants were also identified through publicly available profiles and through snowball sampling. Those individuals were emailed directly with an invitation to participate. All participants were invited to complete a survey where they were asked about the specific PROM and PREM instruments they used, their uses (clinical care, evaluation, research), modes of administration and challenges associated with their use. At the end of the survey, participants were asked if they wished to be contacted for an interview where their experiences with PROMs and PREMs would be explored in more depth. Data from those interviews were used in this study. Interviews were conducted between April and July 2022. The interview was focused on understanding the participant’s experiences with PROMs and PREMs, with an emphasis on the barriers and facilitators to implementing PROMs and PREMs in pediatrics. Interviews were conducted virtually using Zoom software and lasted between 30 and 45 min. They were audio-recorded and transcribed verbatim. Verbal informed consent was obtained from each participant prior to the start of the interview.

### Data analysis

Analysis was performed using NVIVO (QSR International) qualitative data analysis software. We used an inductive, conventional content analysis approach, as described by Hsieh & Shannon (2015) whereby one researcher (EM) generated a codebook iteratively as she read and re-read the interview transcripts [25]. A codebook used barriers and facilitators as primary categories. The data was examined for similarities within each category, and codes were created. Transcript text where the participant discussed a barrier or facilitator to implementation was highlighted and assigned to a code. Codes were combined and new codes were created as the researcher worked through the transcripts. Codes were collated as subcategories within the two major categories, then reorganized by factors affecting implementation. The coding framework was reviewed by a second researcher (SR) to ensure it accurately represented the data. Recommendations for integrating PROMs and PREMs in pediatrics were developed based on data regarding facilitators for implementing or sustaining PROMs and PREMs use.

## Results

A total of 23 interviews from Study 1 and 2 were analyzed in this study. Participant characteristics are summarized in Table 1. We had 2 allied health professionals, 5 researchers, 10 clinician scientists (all physicians), 2 evaluation specialists and 4 AHS administrators. The majority worked within AHS (16/23), with five working in academic institutions, and two working in community clinics. Twenty-one worked exclusively in pediatrics, while two work with both pediatric and adult populations.

**Table 1:** Participant characteristics

Characteristic	Number of participants
**Gender**	
Men	5
Women	18
**Clinical population**	
Child and youth mental health	5
Children with developmental delay/disabilities	5
Pediatric rheumatology	3
Pediatric neurology	2
Children with medical complexity	1
Maternal and child health	1
Neonatology	1
Pediatric chronic pain	1
Pediatric gastroenterology	1
Pediatric nephrology	1
Pediatric physiotherapy	1
Pediatric respirology	1
**Institution(s)**	
Alberta Health Services	6
Community clinic	2
Academic institution	5
Combined academic institution and pediatric hospital	10
**Role(s)**	
Health Service administrator	4
Clinician	2
Clinician scientist	10
Evaluation specialist	2
Researcher	5

We found five main factors that affected implementation of PROMs and PREMs in pediatric settings: 1) Characteristics of PROMs and PREMs; 2) Individual’s beliefs; 3) Administering PROMs and PREMs; 4) Designing clinical workflows; and 5) Incentives for using PROMs and PREMs. Each factor and recommendations for a strong implementation strategy are described in detail below.

### Characteristics of PROMs and PREMs

Participants perceived PROMs and PREMs as being valuable tools, however many had concerns about the properties and characteristics of the measures available for pediatrics. Finding an appropriate PROM or PREM for an intended use was a common concern (e.g., a measure with forms for different age groups, or one with both a self and proxy-report version). Also, finding a child or youth measure for a particular construct of interest was also a challenge, and adult measures had to be borrowed or adapted, affecting their validity.

*“There isn't one single measure that actually covers the age range of youth you're looking at. So, like I've done some studies with kids as young as two up to 18 [years old] about their experiences in hospital. And there's not a great measure that applies across that age range so then you're trying to combine things.” Participant S, Researcher*

Participants also questioned the validity of some of the measures they were using. There were concerns about measures’ cross-cultural validity (e.g., items asking about school and extra-curricular activities not valid in the context of the global pandemic or for different cultures, norms developed in the UK were valid in the Alberta context). There were also concerns about the interpretability of certain items (i.e., whether the child or caregiver truly understands what a question is asking). Related to this, another common concern was the lack of availability of measures in different languages, either because the translations have not been developed, or because they weren’t available within their institution.

An additional challenge, highlighted by clinicians working with pediatric populations with more severe impairments, was that PROMs, especially generic PROMs, were not relevant to their patients. This was problematic from a measurement perspective because the PROMs were not effective in capturing change in the child’s function. More importantly, from a patient and family-centred care perspective, it was observed that this could have a negative effect on the child or parent responding to that PROM.

*“And some of the families that I've worked with have reported that PROMs feel quite negative to them, like when you're asking functional questions about: “Can they do this independently? Do that independently?”... and in my population none of the kids can do any of those things, and I think for parents having to outline that is really quite troubling.” - Participant B, Clinician scientist*

In terms of facilitating PROMs and PREM use, our participants felt that the availability of measures that were short (i.e., clinically feasible), while still valid and reliable, in the languages most often spoken by caregivers and children in their clinics, with questions that are easily understood and interpreted, and with multiple forms for different ages, was important. There was not a clear consensus as to what characteristics of PREMs would facilitate their use. Some participants desiring more standardized and validated PREMs to use across programs, while others suggesting alternative ways of capturing patient experiences such as qualitative interviews, or custom, context-specific surveys.

### Individuals’ beliefs

Participants expressed that getting buy-in from staff was a challenge to implementing PROMs and PREMs. Most commonly, clinicians cited that PROMs and PREMs are an additional burden and could take away from the time a clinician has to spend with patients.

*“From the clinician’s perspective, we've heard that it's very time consuming- that it's going to impact client care potentially because they don't have as much time to spend with the client because they are busy entering data, etc.” – Participant A, Evaluation specialist*

Participants found that automating the PROMs data collection and scoring process could help overcome this barrier. Additionally, patients’ responses should be easily accessible by clinicians, and the data generated by PROMs should provide clinically useful information above and beyond what the clinician would get from their typical clinical interview with the patient.

A second attitudinal barrier was concerns about the potential consequences of measuring patient outcomes. Clinicians’ professional identity might be threatened if PROMs data show their patients are not improving. Also, they may worry that PROMs data will be used to monitor their performance.

“If I'm a therapist I might be wondering is this going to come up in my performance appraisal that you know compared to everybody else in the clinic I'm the least effective person or I'm not at this* benchmark? and that's what we've been trying to let people know is we're not going to be using these measures for that, this is for client care but that's a bit of a hard sell.” – Participant C*,* Administrator*

It was reported that both managers and clinicians were concerned that their programs will lose funding if PROMs data show they are not as effective as they thought. Managers also worried that decision-makers may not appreciate the difference between patient satisfaction scores measured by some programs (which are typically high) and the patient-reported outcomes measured in their own programs, and so they might be disadvantaged in comparisons across programs.

To attempt to address these concerns, participants had three suggestions. First, they felt that there had to be a relationship of trust between administrators and clinical staff, and that administrators should clearly communicate the purpose of using PROMs or PREMs data (e.g., the decisions being made based on the data, and if and how they would be used for managing clinician performance). Second, managers should cultivate an attitude of continuous quality improvement and professional development—where PROMs and PREMs are seen as an extension of this—as a way of mitigating the potential threat to professional identity that could be experienced by clinicians and also increase buy-in. Finally, it was felt that involving staff in the implementation design process was effective for increasing buy-in.

*“We are embedding a culture of ongoing quality improvement, and that culture, along with the structures and processes, has shifted the thinking.” - Participant R, Researcher*

### Administering PROMs and PREMs

The administration of PROMs and PREMs to patients and families (i.e., how they are collected, scored and recorded in a patient’s medical record or quality improvement database) created barriers for participants. Paper and pencil questionnaires were often used by participants, and they were viewed as less desirable than electronic PROMs collection methods. It was reported that with paper questionnaires, respondents would often miss questions, and they are less efficient as someone is required to manually score the PROM and enter data into the patient’s medical record or a database.

*“The biggest challenge was the paper and pencil piece being cumbersome, scoring it right in the moment, you got to measure things, and then tracking it, you know, but the biggest challenge was it was not integrated with the electronic medical record right track it overtime or anything.” – Participant C, Administrator*

Almost all participants felt that having access to fill out PROMs electronically was preferable to paper. Electronic PROMs collection allows for the potential to automate the entire PROMs administration process (i.e., sending PROMs to the patient, collecting responses, automatic scoring, and automatic entry into the patient’s electronic medical record (EMR)). It also provides the opportunity to create graphical representations of scores, which participants felt were appealing to patients and clinicians. Participants liked the option of sending PROMs to patients to complete ahead of their appointment at a time that is convenient for them and felt that they can facilitate PROMs collection in telehealth encounters. The idea of integrating PROMs into the EMR was a highlighted as essential for the long-term successful integration of PROMs into the pediatric health system. However, participants described difficulties related to access to patient portals for EMRs in the pediatric context. For example, EMR system may be set up so that the child’s EMR is shared with a parent, meaning caregivers will have access their child’s responses to PROMs. Another barrier cited was a requirement for official government identification that includes a photo of the child (e.g., a driver’s permit) to verify a child’s identity prior to giving them secure access to their EMR.

*“So with [patient portal] we've been finding that a lot of people aren't signed up for it yet, and it's not necessarily the easiest process because you need to verify that you are the person who owns the chart, right? So, for adults, this is easy, you provide them a driver’s license with a photo image, etc., but for youth it's a little difficult because they don't necessarily have an official ID and things like that, so for that population that's an additional barrier to consider in using a digital chart tool.” – Participant A, Evaluation specialist*

Participants were addressing these challenges through having upfront discussions between the clinician, caregiver, and child about when and how the child’s responses to PROMs will be available to parents. They also suggested that having separate portals for the caregiver and child on the EMR, with the ability for the clinician to control which pieces of data are available to parents and the children would help address this issue.

Flexibility in modes of administration was mentioned as important for equity. Electronic may work for some children and their caregivers, but interview administration may be better for families who have language or technological barriers. It was felt that having a person help with completing PROMs also improved inclusiveness and accessibility by helping people with learning disabilities and language barriers, and also to overcome some of the interpretability issues with the measures. When a child required help completing a measure, it was felt by some to be desirable that health team member assist the child rather than a parent, so that their responses are not influenced by the parent. Additionally, making electronic devices available in clinics for those who are not able to complete them at home or in settings where appointments are not scheduled in advance was suggested if using electronic platforms.

A common theme among participants related to administration of PROMs was the importance for patients and clinicians have access to the patients’ PROM responses. This was an issue in situations where PROM responses go directly into evaluation or research databases, where it’s difficult or not possible for clinicians and patients to view the scores. For clinicians, they felt that PROMs scores gave them extra pieces of information about their patient that help inform decision-making. It was also described to be an incentive for them to actively collect PROMs data within their clinic.

*“When I am seeing somebody in follow-up I've seen multiple times, I'll pull out their patient summary report and I can see the graphs of how they're doing over time and know where to focus: ‘okay, their headaches are better, but the depression is worse, etc.’ and it's really helpful for patients too” – Participant G, Clinician scientist*

When patients see their PROMs responses being used by their clinicians access to their results, it was felt that they were more motivated to engage with PROMs. It was also seen as empowering for patients, because they can, for example, track their own progress over time. Some also saw having access to their scores as a patient right.

*“You have to make PROMs relevant [for patients] so if you ask them to spend 10 minutes on checking off boxes then you need to make sure that they know that it's relevant for you or relevant for the care frame you are building.” – Participant F, Clinician scientist*

### Designing clinical workflows

Designing how PROMs and PREMs would be integrated into clinical workflows was a resource-intensive process. For example, informed decisions had to be made about a data collection platform, how patients will be asked to complete the measures, how the data will be presented to patients and clinicians, and how often to administer the measures. Time and resources were also cited as barriers in terms of designing processes to use the data generated by the measures.

In addition to these design issues, participants in pediatric settings had additional ethical and practical considerations. For example, whether parents have a right to respond to PROMs about their child without their child’s consent, decisions about whose responses should be weighted more heavily in clinical decision-making, whether caregivers have a right to view their child’s responses, and the age at which a child has a right to keep their responses private from their caregivers. In the case of children with cognitive impairments or very young children, they had to decide if a proxy-report alone is sufficient or whether it would be feasible to get a valid rating from a child of their own health.

*“I think with the pediatric populations, you get into this area of consent issues because parents have access to their child's records, and sometimes the clients don't necessarily want their loved ones knowing how they're doing and things like that, but they have access to the chart as well, so there's been some issues that we've been dealing with regards to that.” - Participant A, Evaluation specialist*

A considerable task for participants was choosing the right measures to use. For our participants, this required learning about measurement properties and the available PROMs and PREMs. Some participants also wished for access to a network of PROMs users in their clinical area or a repository of PROMs with information about who is using which measures. Minimizing respondent burden was also a consideration when choosing which measures to use. It was reportedly difficult to balance the desire to gather good information while not overburdening children and parents with lengthy or overly frequent questionnaires.

Designing workflows to obtain adequate response rates was another challenge for participants, more with PREMs but also with PROMs when paper and pencil forms were used and or when participants are sent PROMs after treatment is complete. Response rates were reportedly improved when patients and clinicians have access to the PROMs data. It was also noted that patients and families are motivated to complete PREMs when they see how the data are used to drive improvements in their health service. For example, by public displays outlining the actions taken based on PREMs data.

*“Families, I think you have to look at what's the incentive for them to complete it. Hopefully they understand that PREMs drive changes.” – Participant B, Clinician scientist*

A final concern for participants, particularly clinicians, was to ensure that there are adequate resources and processes in place to follow-up on issues identified by a PROM. For example, referral processes for issues that are outside their scope of practice or urgent concerns, like if a PROM identifies a child at an immediate risk for self-injury. In the child and adolescent mental health field, it was felt that the clinicians’ responsibilities needed to be clearly outlined if a potential for self-injury is identified in a patient completing a PROM remotely (i.e., from outside the clinic).

*“You know, you're asking a patient “tell me how you feel?” and then they tell you “I feel crap,” and then you’re saying “I'm sorry, we don't have the resources to do anything about it.” Right? So I think that is always when you include them in your clinic, you have to think about, you know, are we able to handle this? *I think that's one of the- the things that people sometimes forget* that if you include PREMs or PROMs you have to be able to act on them.” – Participant Q, Clinician scientist*

### Incentives for using PROMs/PREMs

Participants expressed a variety of motivations for using PROMs and PREMs. These basically broke down into 5 categories: policy, demonstrating value to funders, quality improvement, research, and personal incentives.

1. *Improve care at the individual-level* Almost all participants felt that using PROMs improved patient care by enhancing the clinicians’ understanding of the patient as a whole person, and through facilitating shared decision-making.
2. *Enhancing quality of care* Some participants were motivated to use PROMs and PREMs because the data can be useful for making improvements to their services, or to help make decisions about resource allocation. Some clinicians were interested in using their PROMs data as a feedback tool for their own professional growth. PROMs were also seen as beneficial in advocating for additional services for certain populations because they can demonstrate the burden of those conditions.
3. *Demonstrate value* Administrators and clinicians were incentivised to capture PROMs data into order to show the value of their programs, either in anticipation of asking for more funding to expand a program, or for when decisions resource allocation. It was also felt that PROMs could be used to demonstrate the value of a program to its staff, who might feel more satisfied with their work if they see the benefit to their patients.

*“This is only one incentive, but we were developing a resource allocation framework that looked at what aspects of a program should be in place in order for funding to continue or for new funding, for that matter... and one of the measures that we asked for people to include is their PREMs and PROMs. That was- I think an incentive, you know. On a big level has to do with whether a program is funded or not, whether children get the intervention that they need, and I think clinicians care a lot about that.” – Participant F, Clinician scientist*

4) *Policy* There were some instances where PROMs were mandated by funders. For example, funders requiring a patient-reported outcome be reported as a condition of payment for services or to demonstrate the need for a more expensive pharmacological agent.
5) *Research* Within our participants, the primary incentive for using PROMs was for research purposes (e.g., clinical research, registries), with their use within clinical care or evaluation being an additional benefit.

### Recommendations for integrating PROMs and PREMs

Table 2 summarizes the recommendations for the successful integration of PROMs and PREMs into pediatric health settings.

**Table 2:** Recommendations for integrating PROMs and PREMs into the pediatric health system

Recommendations
1. Choose the most appropriate measures for the context-of-use (i.e., should be relevant, reliable, and valid for its intended use). If possible, choose shorter measures, with translations that reflect the populations, with multiple forms to reflect different age groups and proxy/self-report
2. Measures should provide value-added information for clinicians
3. Scores should be available to clinicians and patients and represented in easy to interpret ways when possible
4. Automate PROMs and PREMs workflows using electronic PROMs systems
5. Build in flexibility in administration of measures to enhance inclusiveness and quality of data
6. Have a clear plan to use the data
7. Ensure adequate processes and resources are in place to follow up on PROM and/or PREMs findings
8. Build trust between administrators and clinical staff
9. Cultivate a culture of quality improvement within the team
10. Demonstrate institutional commitment
11. Have a comprehensive educational strategy that addresses all individuals needs (i.e., clinicians, managers, support staff, analysts, patients and families etc.)
12. Engage patients and families and staff in PROMs and PREMs implementation planning
13. Capitalize on potential incentives for PROMs/PREMs use

Many of these recommendations were mentioned as facilitators in previous sections, so here we will focus on describing Institutional commitment, Education and training, Implementation science strategies.

#### Institutional commitment

Participants felt that their organizations should demonstrate that collecting patient-reported measures is a priority. For example, by valuing metrics related to effectiveness over more easily collected metrics, such as wait times for access. Organizations should also provide adequate resources and support to teams implementing these measures. This could include funding to obtain licenses for PROMs which have fees and for access to PROM administration systems that integrate with their EMR. Institutions should support additional personnel required for data collection, analytical support for extracting, analysing, and reporting data, and technical support for adapting PROM systems to their clinical context (adding new PROMs, addressing barriers to EMR integration). It was felt that supporting a dedicated implementation team, with protected time to design and sustain patient-reported measure initiatives, was essential.

*“We do have a few programs that don't have any pre or post measures, or even patient-related experience surveys that go out. That is something that needs to be in the works, but it seems to get always bumped to the bottom of the list, so it's not a prioritized task when it comes to management.” – Participant H, Administrator*

A wish for some participants was that their organization would support a central database of PROMs that are available provincially with information about who is using them and for what purpose. This would facilitate coordination between clinics to be using the same PROMs across different program and jurisdictions in the province and also inform the initial selection of measures.

#### Education and training

Table 3 summarizes the basic education and training required when integrating PROMs and PREMs in pediatric settings. In addition to this initial education, ongoing learning strategies were also suggested, such as supporting community of practices around using PROMs within clinical encounters and also implementation strategy. Public displays for staff, patients and families about PREMs, including examples of how the data has been used to create improvements in services was suggested as a way of sustaining engagement with PREMs.

**Table 3:** Education and training needs for integrating PROMs and PREMs

Roles	Education required	Outcome
Everyone	Importance of patient-reported measures and how they are used at the individual and system level to improve care. This should include concrete examples of how this type of data has been used in the past	Buy-in
	Communicate the strategy for how PROMs and PREMs data will be used	Buy-in and trust
	Basic understanding of the workflows around PROMs and PREMs collection and uploading to chart	Increase quality and rates of response
Clinical staff	Education about the PROMs and PREMs themselves (e.g., what constructs are being measured, what questions are being asked, which types of patients the PROM appropriate for, how to score and interpret scores)	PROMs used and interpreted as intended
	How to introduce and convey importance of PROMs and PREMs to patients and families	Increase patient and family motivation to complete
	How to use information from PROMs in care planning	Increase relevance for clinicians, motivation to collect PROMs
	How to include PROM results in their conversations with patients and families	Increase patient and family motivation to complete
Support staff	Good understanding of PROMs workflows, i.e., how and when to collect PROMs (which PROMs for which patients) and how to upload to the patients’ chart and/or central database. They should be familiar with the PROMs so that they can answer questions from patients that may come up	Increase confidence of support staff, increase quality and rates of response
Managers	Training on how to access and interpret results	Appropriate interpretations of data
Analysts	How to score and interpret PROMs and PREMs	Appropriate interpretations in reporting
Clinical staff and/or implementation teams	How to choose appropriate measures	Measures relevant to the population are chosen
Patient and families	How to fill out measures	Increase quality and rates of response
	Importance of patient-reported measures and how they will be used in their care and at the system level	Increase patient and family motivation to complete

#### Strategies from implementation science

These included having a dedicated implementation team, having a detailed strategy for using PROMs and PREMs data, engaging with stakeholders, and using champions. As previously mentioned, having an implementation team in place was viewed as necessary to initiate and sustain projects to integrate PROMs and PREMs into clinical care. This team should have knowledge of how to choose appropriate measures, knowledge about the PROMs and PREMs used including measurement properties, scoring and interpretation. They should understand how to provide education to staff about the measures and how to interpret scores clinically. They should also provide ongoing support to personnel involved in collecting and using PROMs/PREMs data, and monitoring/evaluation to optimize their use. It was also suggested that they should maintain a central site with information about the PROMs used in the project and other resource materials. Most participants felt that a key part of an implementation strategy should be outlining a detailed purpose and plan for using a PROM and PREM data (e.g., will it be used for clinical care, quality improvement, decision-making, performance monitoring etc.).

Engaging with stakeholders was viewed as important for successful implementation planning. Patients and families should be involved in choosing PROMs and PREMs that reflect their priorities and would be feasible to complete, and in designing workflows that will promote engagement with the measures. Staff should be engaged in designing clinical workflows that will work best in their context, and clinicians should be involved in choosing the PROMs that will enhance their practices.

*“I think if we can get [staff] to feel more connected to the PROMs work they will be more invested in the work because they have some ownership over maybe how they grow, or how they help grow the program, or how they contribute, rather than feeling like they're just a cog in the wheel in the work.” - Participant H, Administrator*

Implementation projects valued having an individual or group of individuals as “opinion leaders” within the clinic or organization who understand how PROMs and PREMs will be used within the clinic and their value in improving care.

*“Our implement*ation* team included clinicians who were really excited and wanted to implement [PROMs]*, and I think having that energy really help to get other clinicians on board, and once they realized how quick it was to complete these measures- like some are like 10 questions long, they started to see the value and benefits to be used with clients in their care.” –* Participant A, Evaluation specialist*

## Discussion

There are distinct efforts within Alberta’s pediatric heath system to implement PROMs and PREMs into the health system at the micro, meso and macro level. For the successful integration of PROMs and PREMs into the pediatric health system, teams planning or evaluating implementation projects need to be aware of the factors that will enable or hinder their implementation. In this paper we have provided a broad overview of the challenges encountered by PROMs and PREMs users within a pediatric health system and some of the strategies that were used to overcome them. We also provided a set of recommendations to guide future implementation designs.

The factors and recommendations discussed by our participants included ethical and practical considerations unique to designing and implementing PROMs and PREMs. The involvement of families in the care of a child adds additional complexities related to consent and privacy. As well, extra consideration is needed when deciding on obtaining and weighting child- versus proxy-reporting on PROMs and PREMs. Haverman et al. (2014) describe a hospital-wide implementation of PROMs in outpatient pediatric settings (‘KLIK program’) for children with chronic illness could provide some potential answers to pediatric specific issues encountered by our participants. For example, Haverman et al. [26] suggest 8 years old as the age at which self-report measures should be administered (versus proxy-report only). In the KLIK program, all parents have access to their child’s PROMs responses, suggesting they did not consider this a privacy issue, which is in contrast to some of our participants. Haverman et al. also suggest that caregivers be should asked to report on their own psychosocial functioning and quality of life using PROMs, which was not described by any of our participants.

Our findings align well with other studies and guidelines for PROMs and PREMs implementation from adult settings. Foster et al. (2018) synthesized the barriers and facilitators to PROMs use across reviews of diverse clinical settings, most of which we described in our findings [22]. One incentive described in both our study and Foster’s was satisfying the demands of an external agency, however, Foster’s findings went further to explain that this external pressure may have a negative influence on the data that users collect and report on, which is something that teams should consider when contemplating this as a strategy for promoting implementation [22]. Another difference was in the idea of preparing patients for PROMs, which was mentioned as one of our recommendations, and which Foster notes was lacking in the studies they reviewed. The International Society for Quality of Life Research published a *User’s Guide to Implementing Patient-Reported Outcomes Assessment in Clinical Practice* [19]. Our findings related to designing clinical workflows and characteristics of PROMs align well with this guidance document, however it is focused on the clinical uses of PROMs, and thus does not touch on some of the health system variables we identified, nor does it comprehensively cover issues specific to pediatric contexts. In addition, our recommendations for education and training align well with the considerations for training clinicians outlined by Santana et al. [27].

Due to the secondary analysis design of this study, we did not use a determinate framework in the data collection or analysis. However, our findings can be mapped onto one such as Damschroder’s Consolidated Framework for Implementation Research (CFIR) [28]. CFIR provides a menu of constructs that have been associated with effective implementation organized into five major domains (the intervention, inner and outer setting, the individuals involved, and the process by which implementation is accomplished) [28]. The characteristics of the PROMs and PREMs and administration challenges can be mapped to the *Intervention Characteristics* domain. Individual’s beliefs, including concerns about the burden of collecting and using the data and knowledge about PROMs and PREMs, can be mapped to the *Characteristics of Individuals* domain. Our factor of designing clinical workflows, including engaging patients, families and staff in the design, would map to the *Process* domain of CFIR. In terms of findings related to the *Inner Setting* domain of CFIR, individuals concern about consequences of measurement map there under *culture* and *implementation climate*, and also concerns about the time and resources needed to integrate PROMs and PREMs (under *available resources*). As well, our recommendations for institutional commitment maps to *organizational incentives and rewards* and our recommendation for education and training maps to *access to knowledge and information*. Some of the incentives we outlined (e.g., PROMs being mandated by funders, and demonstrating value) can be mapped to the *Outer Setting* domain.

### Strengths and limitations

Strengths of this study include our approach to qualitative analysis and our study sample which included individuals from diverse stakeholder groups (i.e., clinicians, administrators, researchers). We produced a rich description of factors affecting PROMs and PREMs implementation in a pediatric health system which adds to the sparse literature about experiences implementing PROMs and PREMs in pediatric real-world settings.

A limitation of this study is that we used secondary analysis of two existing datasets, therefore interviews were not focused solely on identifying barriers and facilitators to PROMs and PREMs use. Related to this, interviews were not conducted using an implementation determination framework. Therefore, some aspects of implementation and use may have been missed. A second limitation of this study is that we did not interview a key stakeholder group: patients and families. Doing so would likely have added additional insights into factors affecting implementation.

The findings of this study can guide teams wishing to integrate PROMs and PREMs into their pediatric health systems in planning an implementation strategy. However, the findings present a broad overview from a pediatric health system, and teams should do a thorough assessment of their own contexts using a determinate framework from implementation science (e.g., CFIR, Integrated framework for Promoting Action on Research Implementation in Health Services-specific assessments) [28, 29].

## Conclusions

The use of PROMs and PREMs in pediatrics has the potential to improve clinical care and include the patient and families’ perspectives into evaluations and decision-making. However, implementing and sustaining the use of PROMs and PREMs in pediatric health settings presents several challenges. We have presented a description of factors to consider when implementing PROMs and PREMs in a pediatric health context, as well as recommendations for planning an implementation strategy. This information will be useful for those planning PROMs and PREMs implementation strategies in pediatric settings.

## Supplementary Information

**Additional file 1**. Interview Guide Study 1.

**Additional file 2**. Interview Guide Study 2.

## Data Availability

The datasets analyzed during this study are not available to protect the privacy of participants.

## Abbreviations

AHS: Alberta Health Services
CFIR: Consolidated Framework for Implementation Research
EMR: Electronic medical record
PFCC: Patient- and family-centred care
PREM: Patient-reported experience measure
PROM: Patient-reported outcome measure
