# Supplementary material for: Factors affecting implementation of patient-reported outcome and experience measures in a pediatric health system
Source: J Patient Rep Outcomes. 2023 Mar 9;7:24. doi: 10.1186/s41687-023-00563-1 (PMC9998780; doi:10.1186/s41687-023-00563-1)
Supplement: Supplementary file 1 — Additional file 1. Interview Guide Study 1. [file 41687_2023_563_MOESM1_ESM.pdf]

Interview Guide – Study 1

1. How do you define PROMs/PREMs?

→ give our definition of PROMs/PREMs here: Patient-reported Outcome Measures (PROMs) are used to assess a patient's health status at a particular point in time (e.x EQ-5D). Patient-reported Experience Measures (PREMs) are used to measure patient's perceptions of their experience while receiving care (e.x HCAHPS).

2. Have you ever heard about the use of prems/patient reported outcome measures in routine clinical care?
3. In your opinion, what skills are needed to use patient reported outcome measures/patient experience measures? (prompt –are there any other skills that you need?)
4. How confident are you that patients will receive good care through the use of PROMs/PREMs?
5. What do you think are the benefits of incorporating PROMs/PREMs in routine clinical care?
6. Are there any incentives for you to incorporate PROMs/PREMs in routine clinical care? If yes, what are they?
7. On a scale of 1-10 and 10 being very important, how important do you think it is for you personally to incorporate PROMs/PREMs in routine clinical care?

If participant uses PROMs/PREMs:

1. How do you use PROMs/PREMs in your organization?
2. How are they administered in your clinic/organization?
3. Have there been any barriers to implementing PROMs/PREMs?
4. What has been your experience with using PROMs/PREMs?
5. Why do you use PROMs/PREMs?
6. What resources/support is required for you to continue using PROMs/PREMs?

If participant doesn't use PROMS/PREMS

1. Would you be interested in using PROMs/PREMs?
2. Are there any current barriers to using PROMs/PREMs?
3. What resources/support are required for you to use PROMs/PREMs?
