# Supplementary material for: Factors affecting implementation of patient-reported outcome and experience measures in a pediatric health system
Source: J Patient Rep Outcomes. 2023 Mar 9;7:24. doi: 10.1186/s41687-023-00563-1 (PMC9998780; doi:10.1186/s41687-023-00563-1)
Supplement: Supplementary file 2 — Additional file 2. Interview Guide Study 2. [file 41687_2023_563_MOESM2_ESM.pdf]

Interview Guide – Study 2

Demographics

1. What is your role in your organization?

2. Can you tell me about the clients or populations that your program or clinic serves and the clinical staff you have?

Personal attitudes towards use of PROMs/PREMs

1. We are working from a definition of **Patient-reported Outcome Measures** (PROMs) are used to assess a patient's health status at a particular point in time, this could be a screening tool, a measure of health related quality of life, a symptom inventory or functional status measure (e.x EQ-5D).

   Patient-reported Experience Measures (PREMs) are used to measure patient's perceptions of their experience while receiving care (ex HCAHPS).

   In pediatric care, we also include PROMs filled out by caregivers.

1. How do you define PROMs/PREMs?
   - *Prompts:* Is that similar or different to the way that we define PROMs/PREMs?
   -
2. *[ask if not clear]* Do you use PROMs/PREMs in pediatrics at present?

3. What do you think are the benefits of incorporating PROMs/PREMs in routine clinical care?
   - *Prompts:* Is patient care improved through the use of PROMs/PREMs?

If participant uses PROMs/PREMs

Process questions

1. How do you use PROMs/PREMs in your organization?
   – *Prompts:* For research, quality improvement/evaluation or for clinical care?

2. How are PROMs administered in your clinic/organization?
   – *Prompts: how and when do patients fill them out?*

3. Is that information recorded into the patient's chart?

4. Do clinicians use the information from the PROMs in providing care?

Experience questions

5. What has been your experience with using PROMs/PREMs?

6. What prompted you to start using PROMs/PREMs routinely?

7. Are there incentives for you to incorporate PROMs/PREMs in routine clinical care? If yes, what are they?

Barriers/Facilitators

8. What challenges or obstacles did you have to overcome in order to implement PROMs/PREMs?
   - *Prompts: Are there ongoing issues related to PROMs/PREMs use?*

9. What resources/support are required for you to continue using PROMs/PREMs?
   - *Prompts: What facilitators would help you continue to use PROMs*
   - *Examples: staff training, technical support, user-friendly platforms*

10. In your opinion, what skills are needed to use patient reported outcome measures/patient experience measures in routine clinical practice?
    - *Prompts: If we were to design a PROMs training program, what should be included?*
    - What skills/knowledge do clinical staff need?
    - What skills/knowledge does management/administration need?
    - What skills/knowledge does clerical staff need?
